# Laboratory-Confirmed Avian Influenza A(H9N2) Virus Infection, India, 2019

**DOI:** 10.3201/eid2512.190636

**Published:** 2019-12

**Authors:** Varsha Potdar, Dilip Hinge, Ashish Satav, Eric A.F. Simões, Pragya D. Yadav, Mandeep S. Chadha

**Affiliations:** National Institute of Virology, Pune, India (V. Potdar, D. Hinge, P.D. Yadav, M.S. Chadha);; Mahan Trust Melghat, Amravati, India (A. Satav);; University of Colorado School of Medicine, Aurora, Colorado, USA (E.A.F. Simões)

**Keywords:** influenza, viruses, avian influenza, H9N2, respiratory infections, India, zoonoses

## Abstract

A 17-month-old boy in India with severe acute respiratory infection was laboratory confirmed to have avian influenza A(H9N2) virus infection. Complete genome analysis of the strain indicated a mixed lineage of G1 and H7N3. The strain also was found to be susceptible to adamantanes and neuraminidase inhibitors.

Low-pathogenicity avian influenza A(H9N2) viruses have a wide host range, and outbreaks in poultry have been recorded since the 1990s in China (*1*). In India, avian specimens indicated no serologic evidence of H5N1 and H9N2 during 1958–1981 (*2*); however, 5%–6% persons with direct exposure to poultry had H9N2 antibodies (*3*). Human cases of influenza H9N2 virus infection have been observed in Hong Kong, China, Bangladesh, and Pakistan (*4*–*7*).

An institutional review board approved an ongoing community-based surveillance in 93 villages of Korku tribes in Melghat District, Maharashtra State, India, to determine incidence of respiratory syncytial virus (RSV)–associated deaths among children <2 years of age. A total of 2,085 nasopharyngeal swabs from children with severe or fatal pneumonia were transported to India’s National Institute of Virology to test for influenza, RSV, and other respiratory viruses. A nasopharyngeal swab from a 17-month-old boy received on February 12, 2019, tested positive by PCR for influenza A(H9N2) virus.

The child, a resident of Melghat, had fever, cough, breathlessness, and difficulty feeding for 2 days after illness onset on January 31, 2019. His high intermittent grade fever had no diurnal variation and no association with rash or mucocutaneous lesions. Examination revealed a conscious, restless child with a respiratory rate of 48 breaths/min and lower chest wall in-drawing with intermittent absence of breathing for >20 seconds. He was fully immunized for his age, with bacillus Calmette–Guérin, diphtheria, hepatitis B, poliovirus, and measles vaccines. Both length and weight for age were less than −3 SD. History of travel with his parents to a local religious gathering 1 week before symptom onset was elicited. The father had similar symptoms on return from the gathering but could not undergo serologic testing because of his migrant work. No history of poultry exposure was elicited. The child received an antibacterial drug and antipyretics and recovered uneventfully.

We tested the clinical sample using duplex real-time PCR for influenza A/B, H3N2, and 2009 pandemic H1N1 viruses; RSV A/B; human metapneumovirus; parainfluenza virus types 1–4; rhinovirus; and adenovirus. The sample was strongly positive for influenza A virus (cycle threshold value 20) but negative for seasonal influenza viruses and all respiratory viruses. Real-time PCR analysis for avian influenza viruses H5N1, H7N9, H10N8, and H9N2 revealed positivity for H9N2 virus (cycle threshold value for H9 was 25). We confirmed this result by sequencing the matrix (M) and hemagglutinin (HA) genes of the isolate, designated A/India/TCM2581/2019/(H9N2); the M gene (260 bp) had 97.27% nucleotide identity with A/chicken/India/99321/2009(H9N2), and the HA gene (225,478 bp) had 96.93% nucleotide identity with A/chicken/India/12CL3074/2015(H9N2). 

We then generated whole-genome sequences by using the Miniseq NGS Platform (Illumina, https://www.illumina.com) and a de novo assembly program (CLC Genomics Software 10.1.1 [*8*]). We used MEGA7 (https://megasoftware.net) with a Tamura-Nei nucleotide substitution model including 1,000 replicates bootstrap support (*9*) for evolutionary analysis of 8 genes of A/India/TCM2581/2019/(H9N2) (submitted to GenBank under accession nos. MK673893–900). The HA, neuraminidase, and nucleoprotein gene phylogeny of A/India/TCM 2581/2019/(H9N2) grouped with the dominant G1 lineage (h94.1.1) and clustered with poultry strains from India and human strains from Bangladesh (Figure). The M, nonstructural, polymerase basic 1, polymerase basic 2, and polymerase acidic genes were related to an H7N3 isolate from Pakistan (*10*) (Appendix Figure 1). We confirmed that the A/India/TCM2581/2019(H9N2) strain had low pathogenicity, showing a KSKR/GLF amino acids motif at the cleavage site of HA (335−341 [H9 numbering]). We observed 6 potential glycosylation sites (11, 87, 123, 280, 287, and 472 [H9 numbering]) and loss of 2 sites (208 and 218 [H9 numbering]) in the HA gene of A/India/TCM2581/2019(H9N2) with respect to G1 viruses. 

**Figure:**
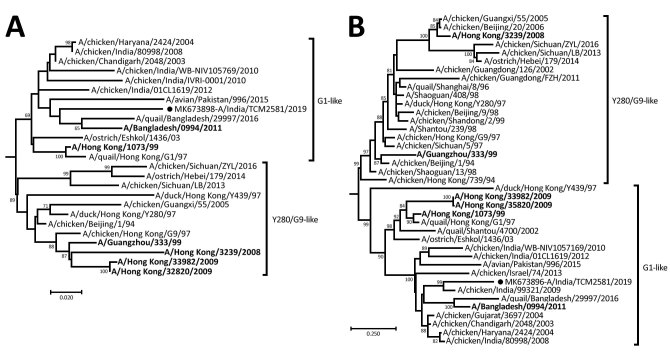
Phylogenetic tree of hemagglutinin gene (A) and neuraminidase gene (B) gene of influenza virus A/India/TCM 2581/2019(H9N2) from India (black circle) and reference strains. The numbers above the branches are the bootstrap probabilities (%) for each branch, determined by using MEGA 7.0 (https://megasoftware.net). Human cases from other countries are in bold. Scale bars indicate nucleotide substitutions per site.

The virus was susceptible to adamantanes with S31 and to neuraminidase inhibitor with R292 and E119 (N2 numbering) (*11*). A/India/TCM2581/2019(H9N2) had Q226L and I155T in HA gene, which promote the human receptor binding. Compared with G1 vaccine strain A/Hong Kong/1073/99, the study strain had multiple mammalian-specific mutations that already exist in poultry-adapted H9N2. The study strain had amino acid changes R207K, H436Y, and M677T in the polymerase basic 1 gene; A515T in the polymerase acidic 1 gene; N30D, T215A, and T139A (all H3 numbering) in the matrix 1 gene; and P42S in the nonstructural 1 gene, all of which are known to be associated with mammalian host specificity and increased virulence in ferrets and mice (*12*). Known markers for virulence and transmission (E627K and D701N) in the polymerase basic 2 gene in the study strain were absent (Appendix Table 1).

Bayesian evolutionary analyses using BEAST version 1.8.1 (*13*) of the HA gene of H9N2 poultry strains from India indicated 3 clusters of multiple introductions at the estimated node age of 2000–2001 (Appendix Figure 2). Human strain A/India/TCM2581/2019(H9N2) and the other poultry viruses from India evolved with 5.163 × 10^–3^ substitutions/site/year.

In conclusion, multiple introductions of H9N2 viruses in poultry have been observed in India. The identification of a human case of H9N2 virus infection highlights the importance of systemic surveillance in humans and animals to monitor this threat to human health.

Appendix Additional information about laboratory-confirmed avian influenza A(H9N2) virus infection, India, 2019.
