# Supplementary material for: Laboratory-Confirmed Avian Influenza A(H9N2) Virus Infection, India, 2019
Source: Emerg Infect Dis. 2019 Dec;25(12):2328–30. doi: 10.3201/eid2512.190636 (PMC6874269; doi:10.3201/eid2512.190636)
Supplement: Appendix — Additional information about laboratory-confirmed avian influenza A(H9N2) virus infection, India, 2019. [file 19-0636-Techapp-s1.pdf]

Article DOI: https://doi.org/10.3201/eid2512.190636

Laboratory-Confirmed Avian Influenza A(H9N2) Virus, India, 2019

Appendix

Appendix Table. The study virus showing multiple mammalian specific mutations in all eight genes reported earlier

Gene	Substitution	Function	A/India/TCM2581/2019
PB2	E627K	Virulence and transmission ofH5N1 in mammals; enhanced polymerase activity; mammalian host adaptation	E627
	D701N, K147T, M147L, K339T, A588T/I	Polymerase activity in mammalian cells of avian H5N1 mammalian cells; polymerase activity; and virulence by regulating the cap binding activity	D701, M147, K339, A558V
PB1	R207K, H436Y, M677T	Polymerase activity in mammalian cells; polymerase activity and virulence in mallards, ferrets and mice; virulence-related mutation	R207K, H436Y, M677T
PA	A515T	Polymerase activity in mammalian cells	A515T
HA (H3 numbering)	Q226L, I155T, H183N, A190V	Q226L promote the affinity of avian influenza viruses for human type receptors	Q226L, I155T, H183, A190
	HA1/HA2 cleavage site	Polybasic amino acid indicates high pathogenicity	KSKR/GLF
NA (N2 numbering)	49–68 deletion (A/Gs/Gud1/1996)	Enhance virulence in mouse	No deletion
	V116A, E119A/G/V, I221M/V/L/K/R, R292K, H274Y	Reduced susceptibility to zanamivir, oseltamivir and/or peramivir	V116, E119, I221, R292, H274
M1	N30D, T139A, T215A	Mammalian host specific markers, virulence related	N30D, T139N, T215A
	V15I	Mammalian host specific markers; common substitution in H5N1 exhibiting high virulence in mice	V15I
M2	L55F	Mammalian host specific markers, virulence related	L55
	S31N/G	Amantadine resistance	S31
NS1	P42S, F103L, M106I, PL motif	Increased virulence in mice; virulence related “(ESEV, EPEV, or KSEV)”	P42S, F103, M106, KPEV

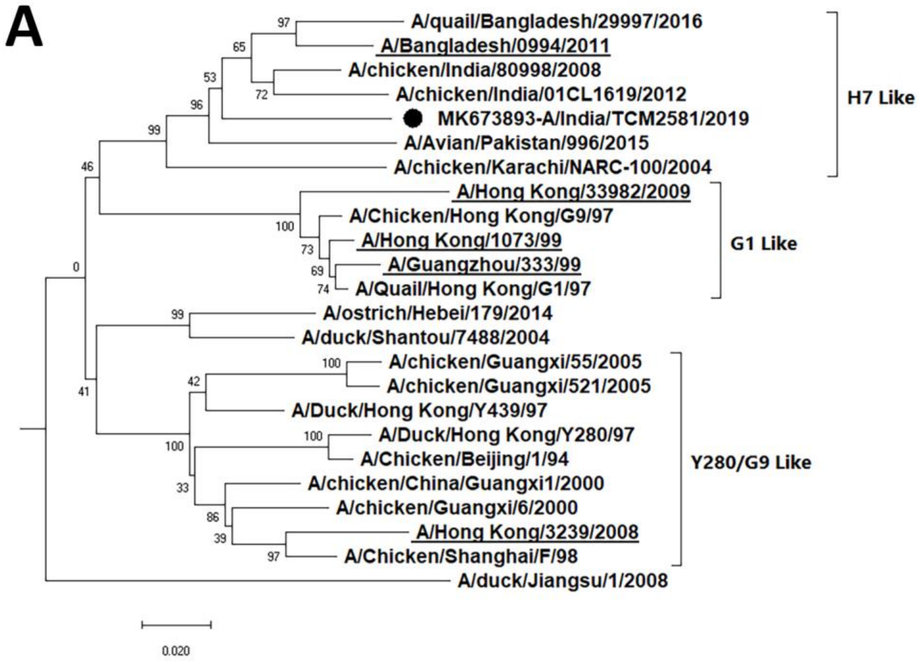
A
A/quail/Bangladesh/29997/2016
A/Bangladesh/0994/2011
A/chicken/India/80998/2008
A/chicken/India/01CL1619/2012
MK673893-A/India/TCM2581/2019
A/Avian/Pakistan/996/2015
A/chicken/Karachi/NARC-100/2004
A/Hong Kong/33982/2009
A/Chicken/Hong Kong/G9/97
A/Hong Kong/1073/99
A/Guangzhou/333/99
A/Quail/Hong Kong/G1/97
A/ostrich/Hebei/179/2014
A/duck/Shantou/7488/2004
A/chicken/Guangxi/55/2005
A/chicken/Guangxi/521/2005
A/Duck/Hong Kong/Y439/97
A/Duck/Hong Kong/Y280/97
A/Chicken/Beijing/1/94
A/chicken/China/Guangxi1/2000
A/chicken/Guangxi/6/2000
A/Hong Kong/3239/2008
A/Chicken/Shanghai/F/98
A/duck/Jiangsu/1/2008
H7 Like
G1 Like
Y280/G9 Like
0.020

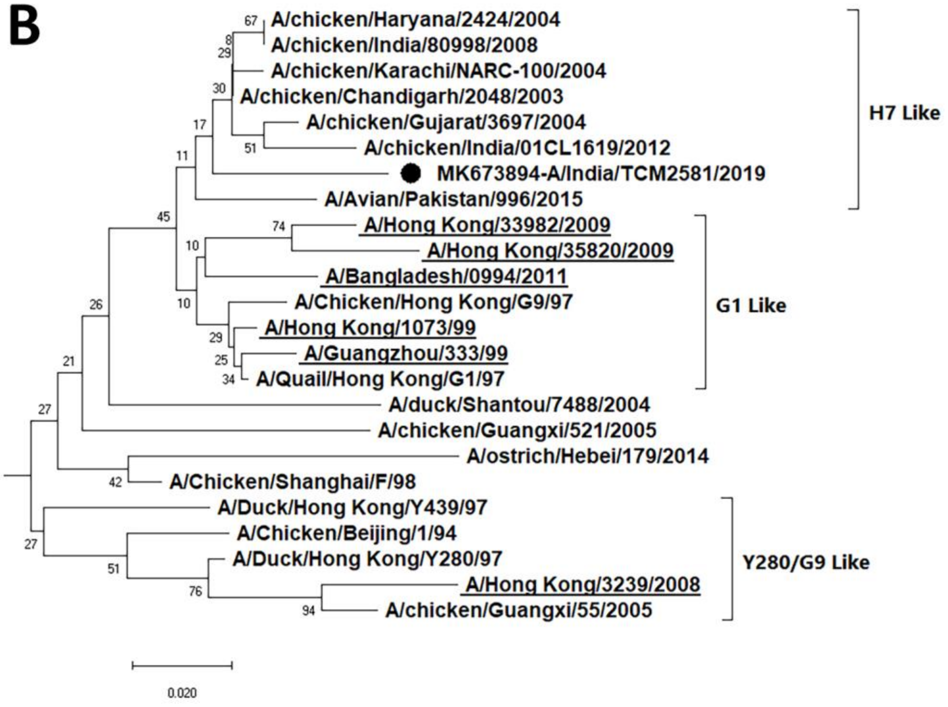
B
A/chicken/Haryana/2424/2004
A/chicken/India/80998/2008
A/chicken/Karachi/NARC-100/2004
A/chicken/Chandigarh/2048/2003
A/chicken/Gujarat/3697/2004
A/chicken/India/01CL1619/2012
MK673894-A/India/TCM2581/2019
A/Avian/Pakistan/996/2015
A/Hong Kong/33982/2009
A/Hong Kong/35820/2009
A/Bangladesh/0994/2011
A/Chicken/Hong Kong/G9/97
A/Hong Kong/1073/99
A/Guangzhou/333/99
A/Quail/Hong Kong/G1/97
A/duck/Shantou/7488/2004
A/chicken/Guangxi/521/2005
A/ostrich/Hebei/179/2014
A/Chicken/Shanghai/F/98
A/Duck/Hong Kong/Y439/97
A/Chicken/Beijing/1/94
A/Duck/Hong Kong/Y280/97
A/Hong Kong/3239/2008
A/chicken/Guangxi/55/2005
H7 Like
G1 Like
Y280/G9 Like
0.020

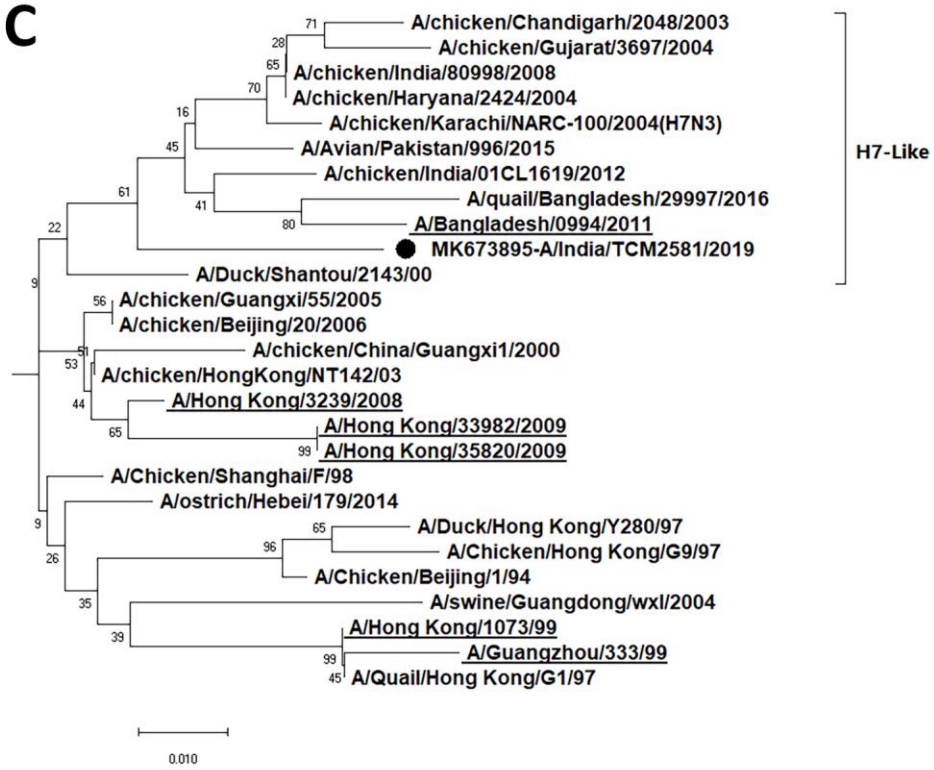
C
A/chicken/Chandigarh/2048/2003
A/chicken/Gujarat/3697/2004
A/chicken/India/80998/2008
A/chicken/Haryana/2424/2004
A/chicken/Karachi/NARC-100/2004(H7N3)
A/Avian/Pakistan/996/2015
A/chicken/India/01CL1619/2012
A/quail/Bangladesh/29997/2016
A/Bangladesh/0994/2011
MK673895-A/India/TCM2581/2019
A/Duck/Shantou/2143/00
H7-Like
A/chicken/Guangxi/55/2005
A/chicken/Beijing/20/2006
A/chicken/China/Guangxi1/2000
A/chicken/HongKong/NT142/03
A/Hong Kong/3239/2008
A/Hong Kong/33982/2009
A/Hong Kong/35820/2009
A/Chicken/Shanghai/F/98
A/ostrich/Hebei/179/2014
A/Duck/Hong Kong/Y280/97
A/Chicken/Hong Kong/G9/97
A/Chicken/Beijing/1/94
A/swine/Guangdong/wxl/2004
A/Hong Kong/1073/99
A/Guangzhou/333/99
A/Quail/Hong Kong/G1/97
0.010

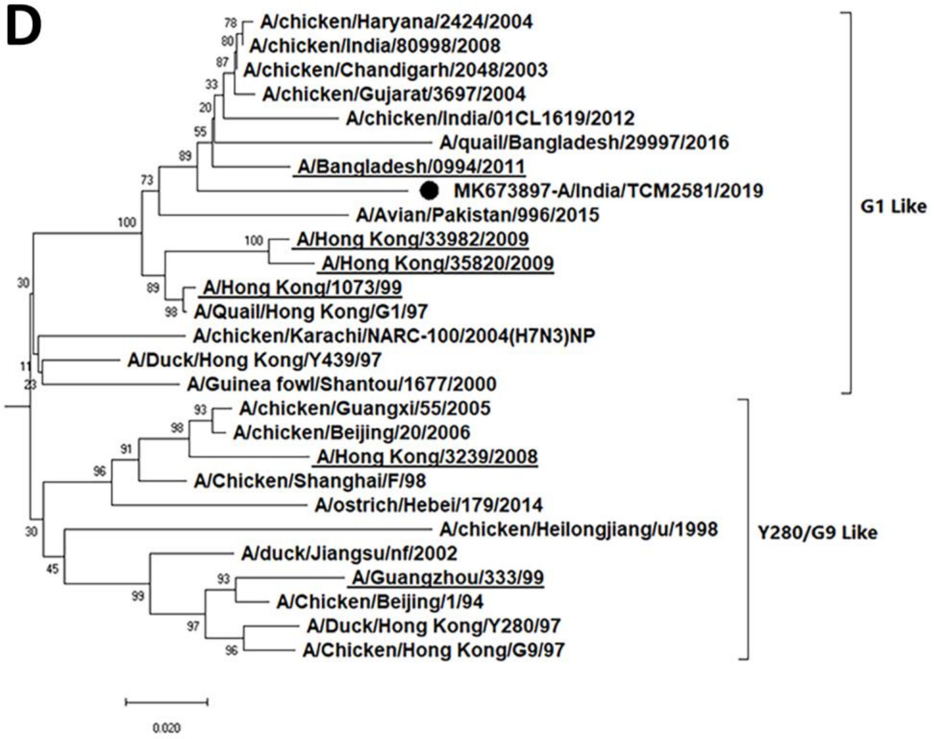
D
A/chicken/Haryana/2424/2004
A/chicken/India/80998/2008
A/chicken/Chandigarh/2048/2003
A/chicken/Gujarat/3697/2004
A/chicken/India/01CL1619/2012
A/quail/Bangladesh/29997/2016
A/Bangladesh/0994/2011
MK673897-A/India/TCM2581/2019
A/Avian/Pakistan/996/2015
A/Hong Kong/33982/2009
A/Hong Kong/35820/2009
A/Hong Kong/1073/99
A/Quail/Hong Kong/G1/97
A/chicken/Karachi/NARC-100/2004(H7N3)NP
A/Duck/Hong Kong/Y439/97
A/Guinea fowl/Shantou/1677/2000
G1 Like
A/chicken/Guangxi/55/2005
A/chicken/Beijing/20/2006
A/Hong Kong/3239/2008
A/Chicken/Shanghai/F/98
A/ostrich/Hebei/179/2014
A/chicken/Heilongjiang/u/1998
A/duck/Jiangsu/nf/2002
A/Guangzhou/333/99
A/Chicken/Beijing/1/94
A/Duck/Hong Kong/Y280/97
A/Chicken/Hong Kong/G9/97
Y280/G9 Like
0.020

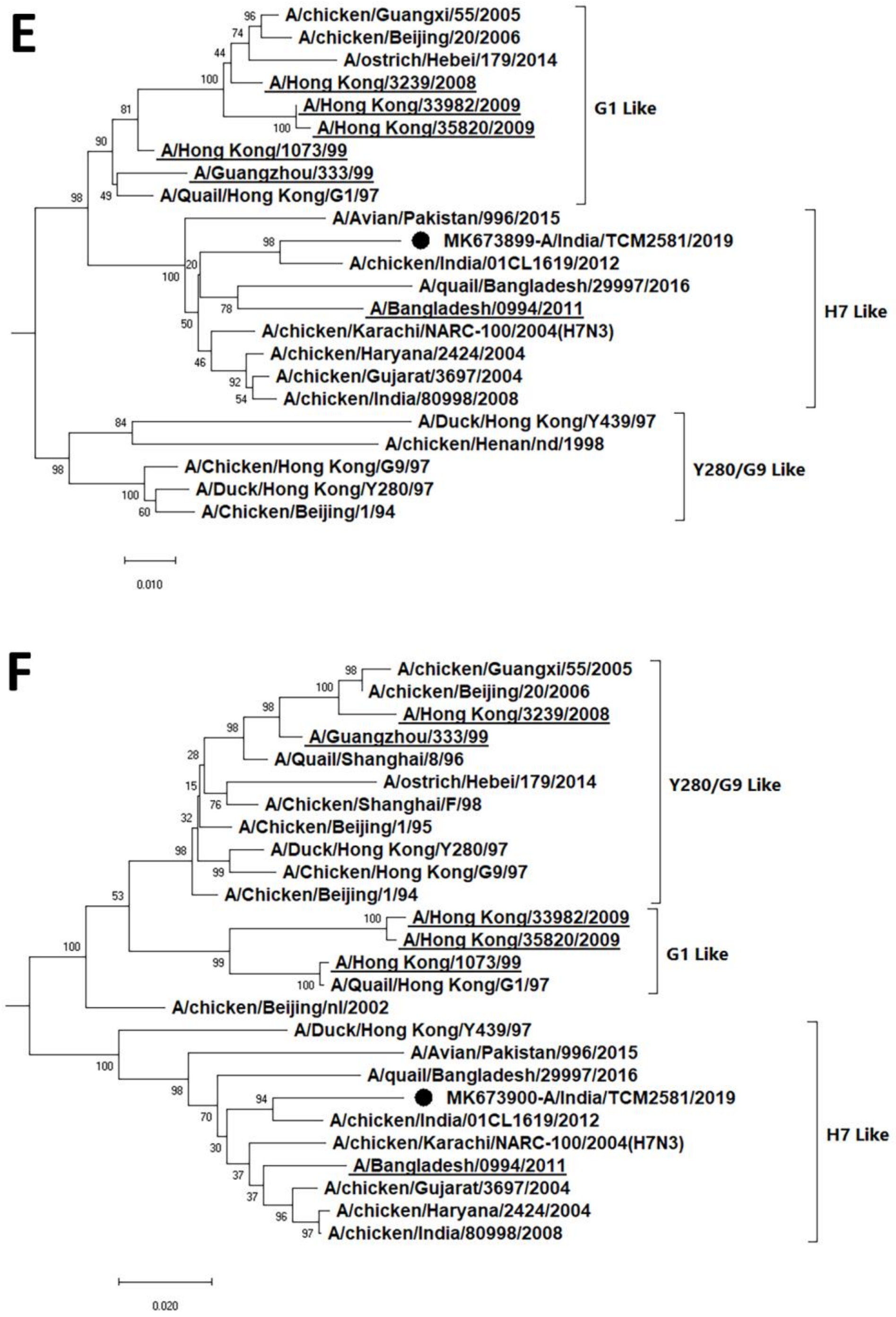

Appendix Figure 1. Phylogenetic tree of six internal gens of study virus A/India/TCM 2581/2019 (H9N2). A) Polymerase basic 2 gene (PB2). B) Polymerase basic 1 gene (PB1). C) Polymerase gene(PA). D) Nuclear Protein gene (NP). E) Matrix gene (M). F) Non structure gene (NS). The numbers above the branches are the bootstrap probabilities (%) for each branch, determined using the MEGA software (version 7). The studied strain is marked with a black dot and the human cases from other countries were underlined.

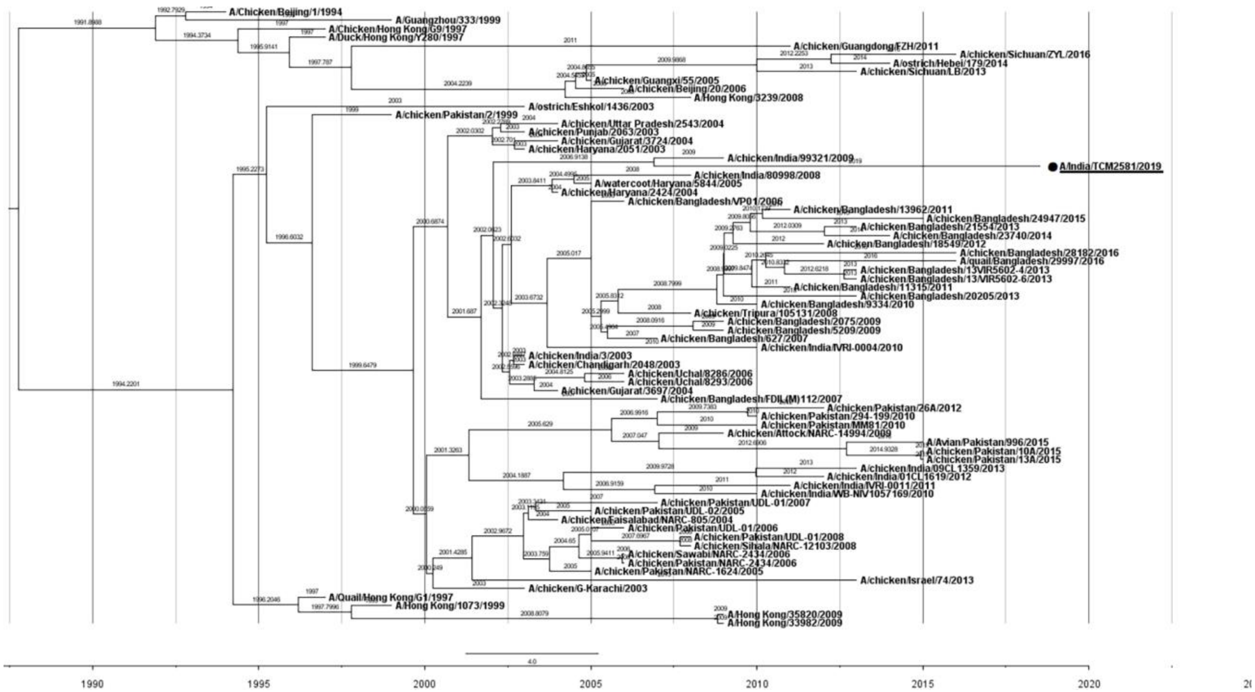

Appendix Figure 2. Bayesian evolutionary tree of Influenza A(H9N2) virus based on the nucleotide sequence of the HA gene. The study virus A/India/TCM 2581/2019 (H9N2) is underlined and marked with a black dot. The evolutionary years are noted for the node. This Bayesian tree was generated using the BEAST software.
